# Gene-set Analysis with CGI Information for Differential DNA Methylation Profiling

**DOI:** 10.1038/srep24666

**Published:** 2016-04-19

**Authors:** Chia-Wei Chang, Tzu-Pin Lu, Chang-Xian She, Yen-Chen Feng, Chuhsing Kate Hsiao

**Affiliations:** 1 Institute of Epidemiology and Preventive Medicine, College of Public Health, National Taiwan University, Taipei 10055, Taiwan; 2 Bioinformatics and Biostatistics Core, Center of Genomic Medicine, National Taiwan University, Taipei 10055, Taiwan; 3 Department of Epidemiology, Harvard School of Public Health, Harvard University, Boston, MA 02115, U.S.A

## Abstract

DNA methylation is a well-established epigenetic biomarker for many diseases. Studying the relationships among a group of genes and their methylations may help to unravel the etiology of diseases. Since CpG-islands (CGIs) play a crucial role in the regulation of transcription during methylation, including them in the analysis may provide further information in understanding the pathogenesis of cancers. Such CGI information, however, has usually been overlooked in existing gene-set analyses. Here we aimed to include both pathway information and CGI status to rank competing gene-sets and identify among them the genes most likely contributing to DNA methylation changes. To accomplish this, we devised a Bayesian model for matched case-control studies with parameters for CGI status and pathway associations, while incorporating intra-gene-set information. Three cancer studies with candidate pathways were analyzed to illustrate this approach. The strength of association for each candidate pathway and the influence of each gene were evaluated. Results show that, based on probabilities, the importance of pathways and genes can be determined. The findings confirm that some of these genes are cancer-related and may hold the potential to be targeted in drug development.

DNA methylation (DNAm) occurs when a methyl group is added to the cytosine in CpG dinucleotides. The presence of such methyl groups can modify DNA and thus alter gene expression. Because DNAm is more stable than gene expression and can represent long-term environmental influences, it may serve as a good candidate biomarker for disease diagnosis, disease prognosis or therapy response prediction^1^,^2^. Acting as an intermediary between genome sequences, environmental influences and gene expression, DNA methylation is a common epigenetic tool that plays an important role in several processes, including those of development and genomic imprinting, and therefore is linked to risk of various diseases^1^,^3^,^4^.

DNAm patterns depend on whether CpGs locate in CpG islands (CGIs)^5^,^6^,^7^,^8^. CpG islands—defined as upstream CG-rich regions with length greater than 200 bps, GC content greater than 50%, and an observed-to-expected CpG ratio greater than 0.6—are often associated with transcription start sites (TSS)^9^. CGIs may cause gene silencing and transcriptional aberrations, as shown in various types of cancers^7^,^8^,^10^,^11^,^12^. In normal cells, methylation mainly occurs at repetitive elements or intergenic regions, while CpG island promoters of genes are usually less methylated or not methylated at all. In cancer cells, however, methylation at non-coding regions is transferred by DNA methyltransferase to CpG island promoters, causing the CGIs to become hypermethylated. These hypermethylated CGIs are thought to block the transcription of tumor suppressor genes, leading to abnormal cell growth^6^,^11^,^12^.

Another issue of concern in the analysis of DNA methylation, as in any analysis of genetic studies, is the need to incorporate biological information for more intuitive and appropriate interpretations. For instance, since most genes do not work alone, inclusion of known information about relationships among a set of genes can help scientists to better evaluate the effect on disease development of the set of genes working jointly. In their consideration of pathways as gene-sets, most current analyses treat the genes located in the same pathway as independent entities. Examples include the knowledge-based pathway analyses, such as over-representation analysis (ORA) and functional class scoring (FCS), which investigate whether certain genes of interest are differentially expressed in given pathways. These approaches consider the pathway simply as a set of independent units, ignoring possible interaction and regulation between components in the same pathway. To account for the position of a gene in a pathway, sometimes called the *pathway topology*, the Impact Factor was later proposed to measure changes in a given pathway when perturbation occurs^13^. This analysis was further modified to rank pathways^14^. The resulting tool allows the same gene to have multiple functions in different pathways, but does not assume the existence of possible interwoven relationships (cross-talk) between pathways. Identification of susceptible genes that includes simultaneous consideration of both their pathway information and the distinctive roles they play in different pathways^15^, remains a challenging task.

Another concern arising in the analysis of DNA methylation is confounders. Factors underlying observed variability in DNA methylation include tissue type, cell type, age, and gender^16^,^17^,^18^,^19^,^20^. Specifically, several studies have identified CpG sites whose methylation levels were associated with age under a cross-sectional or longitudinal study design^16^,^21^,^22^,^23^, using Infinium HumanMethylation27 BeadChip (Illumina Inc., San Diego, CA, USA) or Infinuim HumanMethylation450 BeadChip (Illumina Inc.). Recently, Florath *et al.*^21^ considered a population-based cohort study to identify, and then later to confirm with an independent cohort, more than one hundred CpG sites with methylation levels significantly associated with age. Therefore, it is not surprising that much emphasis has been placed on the importance of designing studies that use twins or age-matched case-controls to adjust for confounders, before making inferences on the association between CpG methylation and diseases^20^,^23^.

To simultaneously include both CGI status and pathway information in the association analysis, as well as to account for heterogeneity across subjects and possible confounders, we propose functions to model the CGI information and pathway knowledge, under an age-matched case-control study design. We adopt the Bayesian approach for its flexibility in modeling stochastic epigenetic variation, such as random individual effects^5^,^24^ and a probe-specific CGI effect that depends on whether or not the probe locates in a CGI. For the pathway association, our model allows that all genes in the same pathway share a common base effect, but that the overall effect of an individual gene can increase or decrease according to its position in the gene-set, which may be related to, for example, the number of neighboring genes it connects to. Previous Bayesian inference on DNA methylation studies assumed that every CpG locus was exchangeable and thus assigned it an equal effect^25^,^26^. Our proposed model aims to relax this assumption for more general cases. To illustrate the methodology, we consider three cancer studies with 104, 32, and 16 case-control pairs, respectively. The first two applications are of DNA bisulfite methylation profiling from ovarian and lung cancer studies^20^,^27^ and the third is of NGS methylation profiling, by reduced representation bisulfite sequencing (RRBS) of high-grade ductal carcinoma *in situ* (HG-DCIS), from a breast cancer study^28^.

## Results

**Figure 1:**
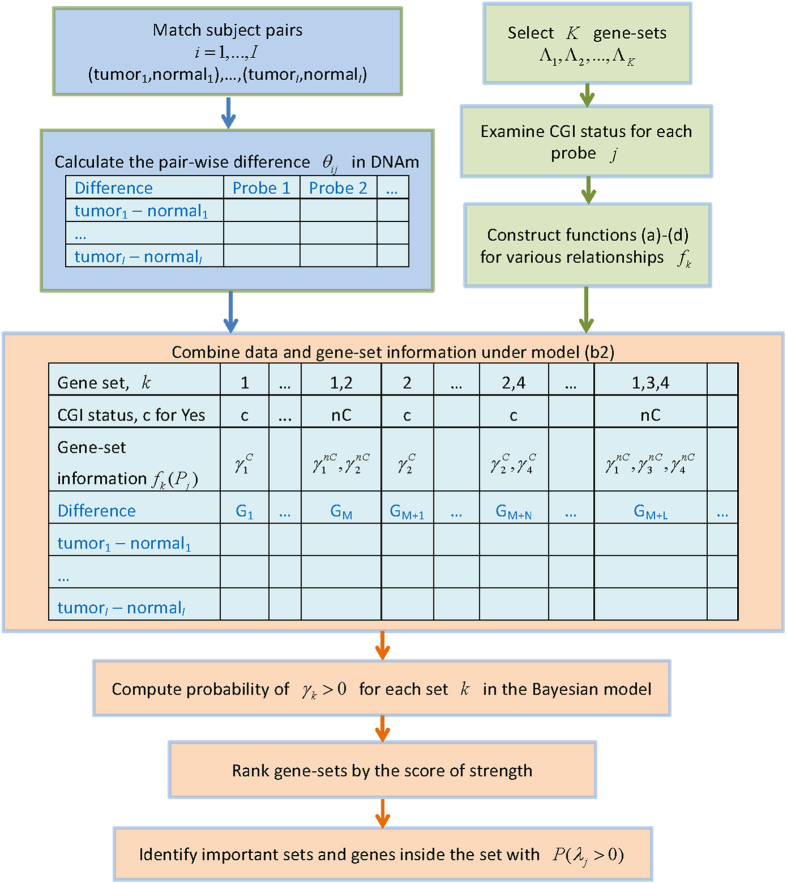
Overview of procedures.

An overview of the procedures, including subject matching, examination of CGI status, and construction of gene-set information, is outlined in Fig. 1. To illustrate how the above model and functions can be applied, we consider first the United Kingdom Ovarian Cancer Population Study (UKOPS)^20^. The DNA methylation data are available from the GEO database (accession number GSE19711) with 27,578 CpGs per sample. The methylation level of each specific CpG site was calculated from the intensity values of methylated and unmethylated DNA beads as a ratio of fluorescent signals, called *β* values. The procedures for data management—outlier detection and removal of batch effect, normalization, and matching—are explained in Supplementary Text S1, available online. To remove the confounding effect due to age, we matched the case-control pairs by restricting the difference in age to be less than three, obtaining 104 case-control pairs for subsequent analysis.

The second application involves methylation profiling from tumor and adjacent normal tissues of 32 patients with lung adenocarcinoma^27^. This study provides a perfect matching design for reducing possible confounding effects. The data can be accessed from NCBI GEO database with accession numbers GSE19804 and GSE49996. For the third application, we consider next-generation sequencing DNA methylation by RRBS from a study of pure high-grade ductal carcinoma *in situ* of 23 breast cancer patients and 5 normal controls^28^. This type of data is uncommon and often of small sample size. Therefore, we match every normal control with more than 1 cancer patient of the same ethinic group and similar age (less than 5 years). This third data set contains 16 pairs. The original data can be downloaded from NCBI GEO database with accession number GSE69994.

### Stratification by CpG island status

**Figure 2:**
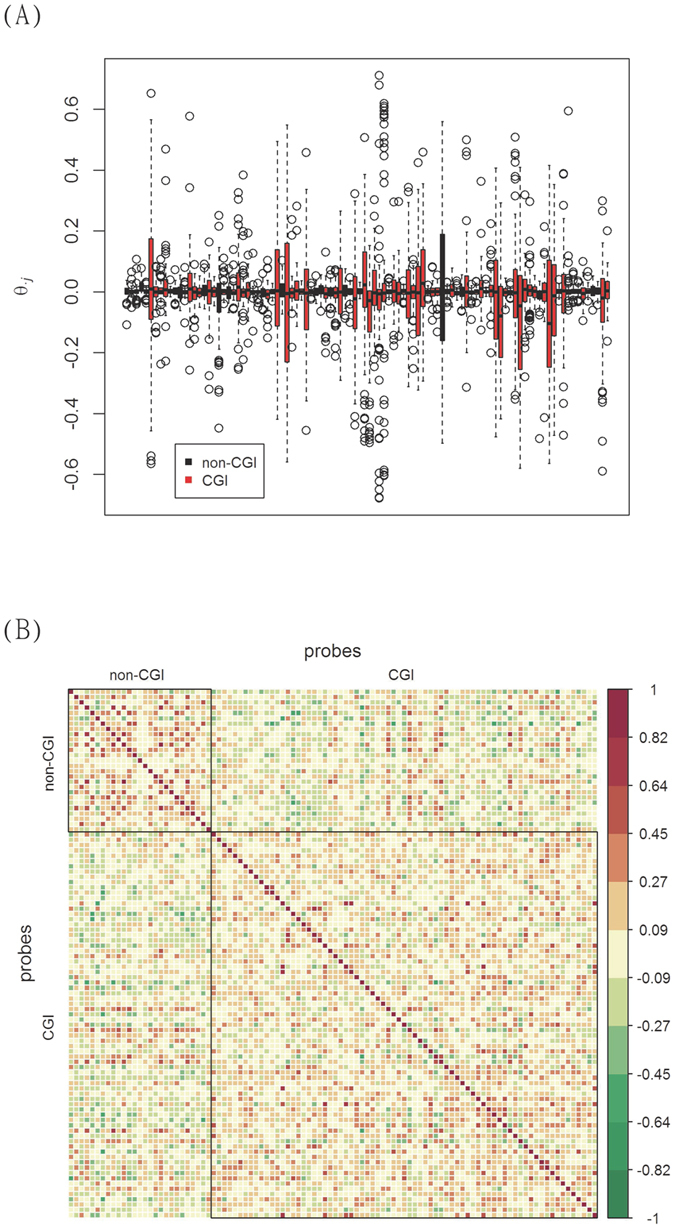
DNAm and CGI status. (**A**) Boxplots of differences in DNAm between matched pairs of ovarian cancer patients (cases) and normal controls for 100 randomly selected probes. The 76 red boxplots are for probes in CGI region; while the 24 black boxplots are for probes not in CGI. Probes in CGI tend to have larger variation in θ_*ij*_, indicating a larger degree of variability in DNAm between cases and controls. (**B**) Correlation plots of θ_*ij*_. The upper left panel contains correlations of θ_*ij*_ from probes not in CGI; while the lower right panel is for probes in CGI. The correlation in each panel is larger, as compared to the correlations in the other two blocks, indicating a greater degree of similarity in θ_*ij*_, the differences in DNAm.

To examine by figures whether DNA methylation varies according to CpG island status, boxplots of the pair-wise differences in DNAm *θ*_*ij*_ from 100 randomly selected probes were constructed (Fig. 2A) for the UKOPS study. This figure included 76 probes located in CGIs (called CGI probes) and 24 located outside (called non-CGI probes). It can be readily observed that the differences in DNAm show larger variability when the probes are located in CGIs (colored in red); while the probes located outside of CGIs (colored in black) tend to have smaller dispersion. In fact, among the original 27,578 probes, the means of the *θ*_*ij*_ were −1.1 × 10^−3^ and −5.8 × 10^−4^, respectively (p < 1 × 10^−7^), supporting the assumption that CGI probes and non-CGI probes are not homogeneous. Moreover, probes of the same CGI status tend to have similar values of *θ*_*ij*_. Figure 2B shows the correlation between non-CGI probes and the correlation between CGI probes. A clear pattern emerges; probes are more alike if they are of the same CGI status. Both Fig. 2A,B support the assumption that the effect of CGI status *β*_*j*_ for probe *j* can be assumed to come from one of two distributions, depending on the CpG island status of the probe. Similarly, the pattern of heterogeneous variation is apparent in the differences in DNAm among the 32 pairs of the lung cancer data. The boxplots of *θ*_*ij*_ from 200 randomly selected probes in CGIs and from 200 not in CGIs show different degrees of variability, as seen in Supplementary Fig. S1a,b. For the NGS methylation data, the number of pairs is only 16, and thus no boxplot is produced.

### Incorporation of biological pathway information

For the UKOPS study, we selected ten competing pathways defined in KEGG (Kyoto Encyclopedia of Genes and Genomes). The ten sets involve 1,675 probes in 795 genes and the ten pathways have been reported to associate with ovarian cancer^29^. Among the ten pathways, four (drug metabolism - cytochrome p450, metabolism of xenobiotics by cytochrome p450, tyrosine metabolism, and arginine and proline metabolism) are associated with the metabolism of xenobiotics or amino acids; while another 4 pathways (focal adhesion, cell cycle, p53 signaling pathway, and oocyte meiosis) are related to cellular processes such as cell communication or cell growth and death. These ten pathways are not of equal size. The tyrosine metabolism pathway is relatively small with 31 genes covered by 57 methylation probes; while the “pathways in cancer” pathway is huge with 303 genes covered by 714 probes. The numbers involved in the other pathways are listed in the first column in Table 1. For the lung cancer DNAm, the number of pairs is only 32 and thus only four signaling pathways were selected: axonal guidance, GNRH, prolacin, and glycosphingolipid biosynthesis. These four pathways contain 103, 58, 46, and 24 genes, respectively. For the 16 pairs matched from the NGS methylation data, we consider only two signaling pathways (mTor and p53) to avoid instability in estimation due to the small sample size. These two pathways contain a total of 2,468 probes in 71 genes.

**Figure 3:**
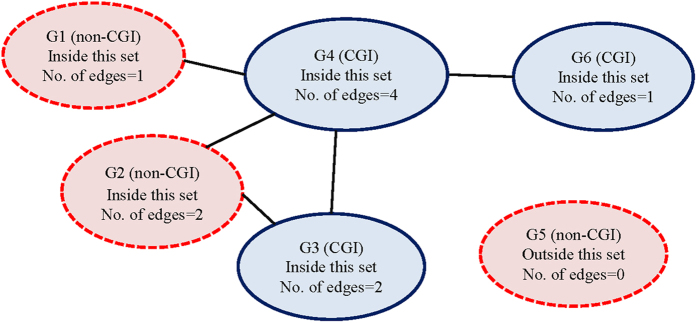
A hypothetical gene-set. Gene nodes in red (G1, G2, and G5) contain no probes in CGIs; while nodes in blue (G3, G4, and G6) contain probes in CGI regions. All gene nodes but G5 belong to this gene-set. The number of edges represents the number of genes connected to it.

To illustrate how to evaluate the pathway effects, we denote first the CGI-dependent effect *β*_*j*_ for each probe *P*_*j*_, where *j* = 1, …, 1,675 for the UKOPS data. Each probe *P*_*j*_ was examined first to see if its corresponding gene *G*_*j*_ falls in the *k*th pathway where *k* = 1, …, 10. Note that the same gene can occur in more than one pathway, i.e. the indicator function *I*_*k*_*(P)*_*j*_ can be 1 for more than one *k*. Figure 3 provides a hypothetical gene-set. In each gene node, its CGI status is 1 (*I*_*k*_*(P)*_*j*_ = 1) if *P*_*j*_ is in a CGI, *E*_*jk*_ is the number of neighbors of gene *G*_*j*_ in the *k*th pathway. No differentiation is made between the incoming and outgoing edges in the current settings. For selecting the best model, we adopt deviance information criterion (DIC), a common measure for Bayesian model selection. Other details of the specifications, computations, and the codes to be used in the R package R2OpenBUGS are listed in Supplementary Text S1.

### Evaluation of pathway effects

To infer the pathway effect *γ*_*k*_, we derived the estimated posterior probability of positive *γ*_*k*_ for each pathway, conditioning on the observed methylation levels θ = *θ*_*ij*_. If the probability *P*(*γ*_*k*_ > 0|θ = (*θ*_*ij*_)) is close to 1, it implies that, on average, genes in this pathway were more hypermethylated in cancer patients. On the other hand, a probability close to zero indicates that *γ*_*k*_ was mostly negative, implying hypomethylation for the genes in the *k*th pathway.

#### UK Ovarian Cancer Population Study

Table 1 lists the probability of hypermethylation Pr(*γ*_*k*_ > 0) among cancer patients for each pathway under different models. The top two pathways, the pathways in cancer and the cell cycle pathway, correspond to the two most extreme probabilities. For the pathways in cancer, the probability was close to zero (e.g., 0.01 under model (b1)), indicating a general pattern of hypomethylation in this pathway. In other words, cancer patients were the least hypermethylated in this group. This pathway consists of several signaling networks (such as the p53 signaling pathway and the cell cycle pathway) related to cancer prognosis, including those of tissue invasion and metastasis, sustained angiogenesis, evading apoptosis, proliferation, failed repair of genes, insensitivity to anti-growth signals, genomic damage, resistance to chemotherapy, and blocking of differentiation. Several genes within this pathway have been reported to show association with ovarian cancer, including Cyclin D1 (*CCND1*), *CDK2*, *ERB2*, and *EGFR*. The cyclins function as regulators of Cyclin-dependent kinases (CDKs) and play an important role in the cell cycle G1/S transition. The four aforementioned genes in this pathway have all been suggested as biomarkers for various cancers.

The probability of the cell cycle pathway was close to 1 (Table 1), implying strong support of hypermethylation for this pathway. Similarly for model (b2), the probability of hypermethylation for probes in CGIs was as high as 0.97. In other words, ovarian cancer subjects hypermethylated to a greater degree than healthy subjects in this pathway. Indeed this pathway regulates all steps in the mitotic cell cycle, including DNA replication (S phase), mitosis (M phase) and the gaps (G1 and G2 phases)^30^,^31^. The pathway includes genes such as *ATM*, *CCND1*, *CDK2*, *CCNB2*, and *CHEK2* that are known to be involved in carcinogenesis.

**Figure 4:**
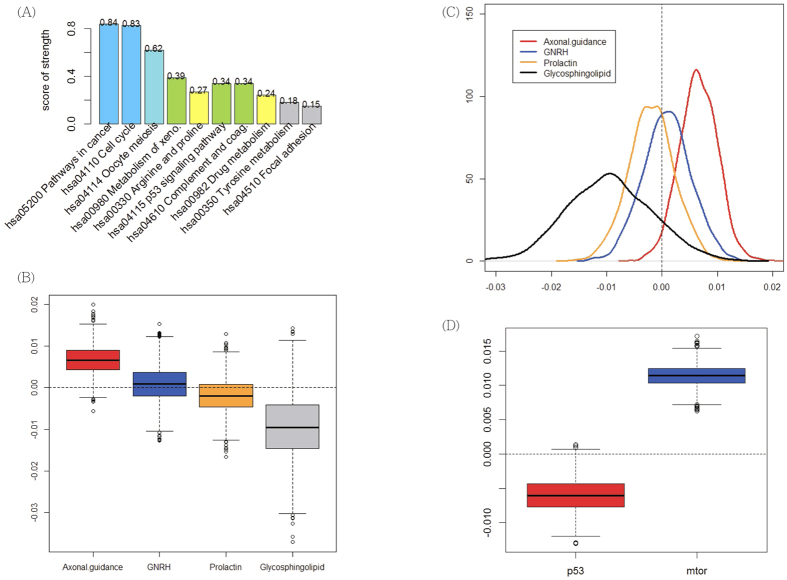
(**A**) Plots of pathway effects. (**A**) Scores of strength for the 10 competing pathways in the UKOPS study. (**B**) Boxplots of posterior samples under each pathway for 32 lung adenocarcinoma patients. A box beyond zero implies a large probability of hypermethylation; while a box below zero indicates a large probability of hypomethylation. (**C**) Probability density plot for each of the four pathway effects. Most of the red curve for the effect of axonal guidance signaling pathway locates in the positive part, indicating a strong hypermethylation effect for this pathway. (**D**) Boxplots of posterior samples in each pathway in the breast cancer study.

An alternative way to compare these pathways is to rank the distances between the probabilities in Table 1 and 0.5 (i.e., no difference). For the purpose of easy interpretation, we rescaled the distance by multiplying by 2, making the distance range from 0 to 1, and called it the score of strength. Figure 4A plots the scores for the corresponding pathways. Clearly the top two pathways stand out. Details of the values are in Supplementary Table S1. To assess which model was most promising for further inference, we considered the DIC (Deviance Information Criterion) under each model. No specific model fitted better than the others, hence the model with the CGI-dependent pathway effect (model (b2)) was selected based on the principle of parsimony and for its better interpretability. The resulting model (b2) was then used to detect influential genes and was compared with other analyses, as discussed in the next section.

#### Lung Adenocarcinoma Study

To evaluate the strength of pathway effect by assigning it a probability, Fig. 4B,C are boxplots and density plots of the four pathway effects, where the axonal guidance signaling pathway ranks as the most important pathway. In this pathway, tumor tissues generally contained hypermethylated CpG sites (with a probability of 0.97); while in the glycosphingolipid biosynthesis signaling pathway, tumor tissues tended to be hypomethylated (with a probability of 0.88). As for the GNRH pathway, the probability that tumor tissues were hypermethylated was estimated to be 0.58, and for glycosphingolipid biosynthesis the probability of hypomethylation was 0.69.

### HG-DCIS Breast Cancer Study

The boxplots in Fig. 4D illustrate the two pathway effects. The effect of p53 pathway is mostly negative, indicating a strong hypomethylation pattern in cases (probability 0.99). In contrast, the mTor pathway shows a moderate hypermethylation pattern among the HG-DCIS patients (probability larger than 0.99). The results not only confirm the importance of these two pathways, but also quantify the effect and its direction of these pathways.

### Disease-related genes

Within the top-ranked pathways, we next examined the differentially methylated probes and genes for disease association with the probe-specific effect *λ*_*j*_. This quantity summarizes the influence from both CGI status and the pathway. If *λ*_*j*_ locates far away enough from zero that either one of the posterior probabilities Pr(*λ*_*j*_ > 0) or Pr(*λ*_*j*_ < 0) is large, say greater than 0.975, then this probe is considered a methylation variable probe. Consequently, its corresponding gene is defined as a differentially methylated gene (DMG). In Bayesian statistical inference, the magnitude of the probability (Bayesian posterior probability, BPP^32^,^33^,^34^) stands for the strength of evidence. Thus, depending on the context, one can select any large value (usually larger than 0.80) as the threshold. Here we used 0.975 simply to focus on the first few leading GO terms.

#### UK Ovarian Cancer Population Study

Under model (b2), 61 DMGs in the pathways in cancer and 14 DMGs in the cell cycle pathway were identified. Among the 61 DMGs identified in the pathways in cancer under model (b2), only 7 have passed the single-marker paired t-test with Bonferroni correction (p < 3×10^−5^); while none among the 14 DMGs in the cell cycle pathway passed the test. Next, we searched PubMed with the query string “human methylation ovarian cancer” and with “human ovarian cancer” and tallied, for each gene that appeared in more than one study, the total number of studies in which a given gene had been reported to be associated with ovarian cancer as of 10 April 2014. Among the 61 DMGs, 32 had been previously reported to associate with ovarian cancer; while 9 among the 14 DMGs in the cell cycle pathway had been previously reported. Examples are *CDKN2A* and *PTEN*, known tumor suppressors in multiple cancers; *EGFR* and *KDR*, known cancer genes; and *RUNX1* and *STAT3*, recently discovered to affect epithelial cancers^35^,^36^,^37^,^38^. Most of the DMGs were detected with probes showing more hypermethylation in controls than in cases (probability larger than 0.975). Exceptions include *CCND1*, *BCL2*, *ERBB2*, and *RARB* genes containing hyper- and hypo-methylation probes, and *FRAP1*, *RUNX1*, *STAT3*, *ATM*, *CCNB2*, *CHEK2*, and *COL6A1* genes containing probes that are more hypermethylated in cases than in controls. Table 2 lists the names of these genes. Some of these genes have been considered as candidates for targeted cancer therapeutics. Our results suggest that DNA methylation can play a role in the development of such drugs. Supplementary Figure S2 demonstrates the location of these genes in the pathway map.

#### Lung Adenocarcinoma Study

**Figure 5:**
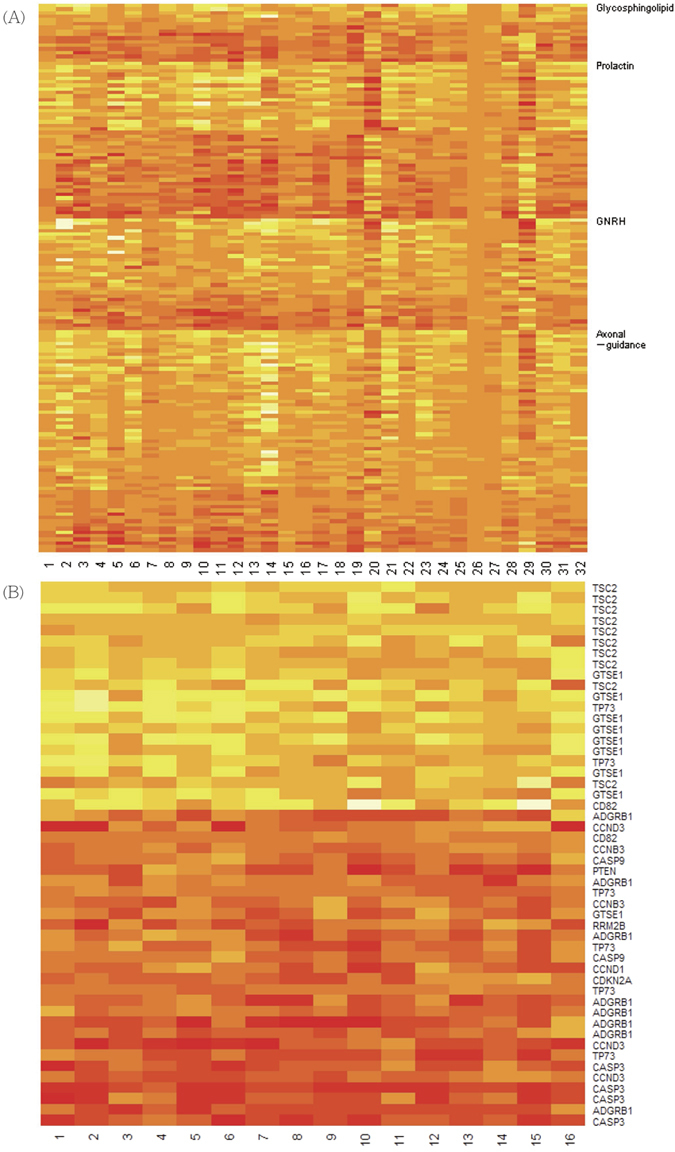
(**A**) Heatmap of differences in DNAm for leading probes. (**A**) These probes are those having the largest probabilities of hypermethylation (yellow) or hypomethylation (red), under each competing pathway in the lung cancer study. (**B**) These probes are the top 50 probes with the largest probabilities of hypermethylation (yellow) or hypomethylation (red) in the p53 pathway.

Figure 5A is the heatmap of *θ*_*ij*_ for the leading genes in each of the four competing pathways. Most genes in the axonal guidance signaling pathway showed a hypermethylation effect in tumor tissue; while most in the prolactin signaling pathway and glycosphingolipid pathway showed hypomethylation. Within each pathway, the DMGs with large probability of hypermethylation and hypomethylation are listed in Table 3 and Supplementary Table S2. Although the major function of the axonal guidance signaling pathway is to regulate the growth of neuronal cells, many studies have reported its association with lung cancer^39^,^40^,^41^. For instance, migration and invasion in lung adenocarcinoma cells can be substantially inhibited through the blockage of the complex *LIMK1*/*PAK4*/cofilin^42^, and methylation changes of *CDKN2A* may interact with *HRAS* to regulate the activity of *MAPK1* in lung adenocarcinoma^43^. Table 3 also lists the expression changes of genes in this pathway. Regarding the other three pathways shown in Table S2, previous reports have indicated their biological impact in lung cancer with expression data as well^44^,^45^,^46^,^47^. Therefore, these results further demonstrate that methylation change is a causative mechanism in regulating the activity of signaling pathways involved in tumor development and growth.

#### HG-DCIS Breast Cancer Study

Table 4 lists gene symbols of probes with large probabilities of hypermethylation and hypomethylation among patients with HG-DCIS. Note that the mTor pathway contains many genes with a large hypermethylation effect; while the p53 pathway carries more genes with a hypomethylation effect. To illustrate the differences in paired DNAm values, Fig. 5B contains a heatmap of 50 probes under the p53 pathway with the largest probabilities of hypermethylation or hypomethylation. It is well-known that the mTor pathway and the p53 pathway play an important role in cancer cell biology. A previous study of breast cancer showed that several genes downstream to *TP53* and/or estrogen receptors were regulated through DNA methylation changes^48^, which agrees with our results that the methylation level of the p53 pathway was affected. Intriguingly, among the hypermethylated genes in the mTor pathway, a previous report has demonstrated that *STK11* was highly methylated in papillary breast carcinoma and its epigenetic dysregulation may be associated with Peutz-Jeghers syndrome^49^.

## Discussion

The advantage of our proposed approach is its ability to start with a list of candidate pathways, and to examine in a unified analysis the effect of each pathway, while controlling for CGI status and pathway structures. Such an approach is useful when researchers have already identified specific competing pathways for further investigation. This approach can quantify the degree of importance among candidate pathways and simultaneously provide methylation information for each gene symbol. Our method can serve as a complementary tool to target pathways or certain gene ontology (GO) terms for further investigation of their molecular function.

Traditional approaches usually perform single-marker tests in the first stage, and next conduct a hypergeometric test for pathway analysis using those gene symbols that have passed the test performed in the first stage. In the second stage, each pathway is tested separately, and no intra-pathway information is considered. We call this *two-stage inference*. Most current methods use online bioinformatics tools and consult databases for annotations. For the purpose of comparison, we considered two procedures. The first applied paired t-tests with Bonferroni correction and then used DAVID for pathway analysis. No pathway was found statistically significant, unless a less stringent significance level of 0.10 was used: pathway of vascular smooth muscle contraction (*p* = 0.067), pathways in cancer (*p* = 0.073), and pathway in prostate cancer (*p* = 0.084). Except for the pathways in cancer, which was identified with our proposed model, these pathways are not associated with ovarian cancer, at least not according to the current literature. In the second comparison, we considered the model with the null pathway effect (a), selected the genes with large probability of hypermethylation or hypomethylation, and then applied DAVID for pathway analyses. A total of 116 genes passed the criterion and two pathways were identified as significant, including the pathways in cancer (*p* < 0.001), and the p53 signaling pathway (*p* = 0.025) based on the modified Fisher exact tests in the DAVID annotation system. Although this procedure did not pick up the cell cycle pathway as our proposed model did, the p53 signaling pathway, which it did identify, can be activated by stress signals, resulting in three major outputs: cell cycle arrest, cellular senescence or apoptosis.

When applying the proposed analysis, other functional forms of pathway topology can be considered. For instance, correlations among genes can replace the number of connecting genes to describe the interaction. Pathways can be extended to networks so that the crosstalk between pathways can be examined, including a network of networks or even the whole interactome^50^. Scientists have observed that some highly connected genes, called hubs, are usually more ancient and give rise to diversified and abundant phenotypes^51^. In that case, the effect of an individual gene may be inversely proportional to its number of links. Therefore, instead of using a simple count of the number of edges linking each gene to the others in a given pathway, an alternative would be to replace the functions in models (c1) and (c2) with analogous functions that model the inverse-degree effect. More details about how to do this in practice can be found in Supplementary Text S1.

## Methods

### Statistical models and pathway effect specifications

In our proposed model for the matched case-control study, each *θ*_*ij*_ denotes the observed difference in DNA methylation levels at the *j* th probe (*P*_*j*_) between the *i*th case-control pair recruited. Each *θ*_*ij*_ follows a normal distribution with mean *μ*_*ij*_, where *μ*_*ij*_ depends on the probe-specific parameter *λ*_*j*_. This *λ*_*j*_ can be decomposed into two parts, one (*β*_*j*_) for the effect of CGI status and the other (Φ) for the pathway effect. Those probes located in CGI share a common effect from the distribution 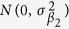; while those probes not in CGI share another common effect from a different distribution 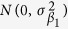. These two distributions allow the pathway effect to depend on CGI status, and allow the variability of the pathway effect to vary when CGI status differs.

For the function of the pathway effect Φ(**γ**; *P*_*j*_, **Λ**), *P*_*j*_ is the probe and **Λ** contains all pathways {Λ_1_, Λ_2_, …, Λ_*K*_} of interest with **γ** = (γ_1_, γ_2_, …, γ_*K*_) as the corresponding parameters. To simplify the notation, we assume that the joint-effect Φ can be partitioned into *f*_*K*_ (*P*_*j*_), where *k* = 1, …, *K*. That is, 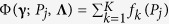.

Each *f*_*K*_ (*P*_*j*_) can be defined according to various scenarios. If each pathway has no effect on methylation, then Φ(**γ**; *P*_*j*_, **Λ**) = 0. This is the null effect model, denoted as model (a). For the case where all genes share the same pathway effect, we consider *f*_*K*_ (*P*_*j*_) = *I*_*k*_(*P*_*j*_) × *γ*_*k*_, and called the CGI-independent constant pathway effect model (b1). However, inside the same pathway, if the effect differs because of CGI status, then the pathway effect would be decomposed into two terms, 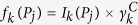 if the probe *P*_*j*_ is in CGI and 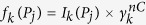 if not. This is called the CGI-dependent constant pathway effect model (b2).

It is possible that the role of a gene depends on its position in a given pathway^13^. For example, the number of its neighboring genes *E*_*jk*_ may imply that this gene is a hub gene and is influential in maintaining the integrity and normal function of the pathway^15^. In network analysis, the number of incoming and outgoing links per node (gene) in a network is called the *degree* or *connectivity*, which can influence the performance of the network. Thus we consider *f*_*K*_ (*P*_*j*_) = *E*_*jk*_ × *γ*_*k*_, and call it the CGI-independent degree effect model (c1). When such an effect is modified by CGI status, then it is assumed 
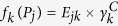
 if the probe *P*_*j*_ is in CGI and 
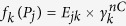
 if not. This is the CGI-dependent degree effect model (c2). The deviance information criterion (DIC) is a common measure in Bayesian analysis for model selection. Here we derived the DIC for each model and selected model (b2) as the final model. All probabilities and inference are based on this model.

### Computations

All the statistical inference was made based on the posterior probability of the parameter of interest, where the probability is evaluated based on samples derived from the Markov chain Monte Carlo method (MCMC). In the above modeling, *γ*_*k*_ measures the strength of association between the *k*th pathway and the disease phenotype. A large value of its posterior probability *P*(*γ*_*k*_ > 0|**θ** = (θ_*ij*_)) suggests that genes in the *k*th pathway tend to be more hypermethylated in cancer patients (cases) than in healthy subjects (controls); while a small value implies more hypomethylation in cases than in controls. Similarly, large values of the posterior probabilities 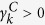 (or 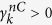) indicate that genes with probes in CGI regions (or non-CGI regions) are more likely to be hypermethylated among the cancer subjects.

To evaluate the degree of association between the gene *Gj* and the phenotype, we examined the association between the *j* th probe and the disease by evaluating the conditional probability *P*(*λ*_*j*_ > 0|**θ** = (θ_*ij*_)). A probability close to one suggests greater hypermethylation among the cases; a value closer to zero suggests hypomethylation.

## Additional Information

**How to cite this article**: Chang, C.-W. *et al.* Gene-set Analysis with CGI Information for Differential DNA Methylation Profiling. *Sci. Rep.* **6**, 24666; doi: 10.1038/srep24666 (2016).

## Supplementary Material

Supplementary Information

## Figures and Tables

**Table 1:** Numbers are the posterior probability of *γ*_*k*_>0 under the specified model for the UKOPS study.

KEGG pathway term (no. of genes/no. of probes)	Constant effect model			Degree effect model		
	(b1) CGI- independent	(b2) CGI- dependent		(c1) CGI- independent	(c2) CGI- dependent	
		Y	N		Y	N
hsa05200 Pathways in cancer (303/714)	0.01	0.02	0.03	0.19	0.10	0.35
hsa04110 Cell cycle (120/308)	0.96	0.97	0.44	0.84	0.89	0.55
hsa04114 Oocyte meiosis (101/211)	0.21	0.14	0.65	0.23	0.18	0.56
hsa00980 Metabolism of xenobiotics by cytochrome P450 (69/122)	0.30	0.31	0.50	0.56	0.17	0.74
hsa00330 Arginine and proline metabolism (53/100)	0.44	0.25	0.70	0.51	0.27	0.68
hsa04510 Focal adhesion (192/418)	0.50	0.45	0.69	0.49	0.45	0.59
hsa04610 Complement and coagulation cascades (64/113)	0.43	0.25	0.41	0.59	0.24	0.54
hsa00982 Drug metabolism - cytochrome P450 (63/111)	0.62	0.46	0.47	0.41	0.66	0.28
hsa00350 Tyrosine metabolism (31/57)	0.49	0.43	0.43	0.48	0.24	0.45
hsa04115 p53 signaling pathway (67/201)	0.32	0.50	0.38	0.57	0.68	0.20

Values closer to 1 imply stronger evidence of hypermethylation in cases than in controls; while values closer to 0 indicate stronger evidence of hypomethylation.

**Table 2:** Gene symbols of the 61 DMGs identified in the pathways in cancer, and 14 DMGs identified in the cell cycle pathway.

Gene symbol	
Previously reported (35 genes)	
In both pathways	*CCND1*^a^*, CDKN2A, CDKN2B, CDK2, CDKN1A, SMAD2*
In pathways in cancer	*APPL1, BAX, BCL2*^a^*, DCC, EGFR, ERBB2*^a^*, EGF23, EGF3, EGF4, EGF5, EGF8, FOXO1, FRAP1*^b^*, IL6, KITLG, LAMB2, MMP2, MMP9, PTEN, PTK2, RARB*^a^*, RUNX1*^b^*, STAT3*^b^*, STAT5A, TGFBR1, WNT2*
In cell cycle pathway	*ATM*^b^*, CCNB2*^b^*, CHEK2*^b^
Previously unknown (33 genes)	
In both pathways	*CCNA1*
In pathways in cancer	*ARNT2, BIRC2, CSF3R, CUL2, CYCS, FASLG, FGF10, FGF12, FGF17, FGF19, FZD10, FZD8, HSP90AB1, JUP, LAMA1, LAMA4, NKX3-1, PIK3CD, PRKCB, PITCH2, RALGDS, RASSF5, RXRB, SMO, SPI1, WNT10A, WNT11, WNT3A*
In cell cycle pathway	*ESPL1, MCM4, TFDP1, YWHAQ*
Previously reported DMGs in other pathways (11 genes)	
	*COL1A1, COL6A1*^b^*, CR1, GSTP1, IGFBP3, KDR, MOS, PGR, PLAU, PPP2R1B, TP73*

^a^These genes contain both hyper- and hypo-methylated probes.

^b^These genes contain probes that are more hypermethylated in cases than in controls.

**Table 3:** Genes with large probabilities of hypermethylation or hypomethylation under the axonal guidance pathway in the lung cancer study.

		Difference in DNAm		Difference in Gene Exp	
		Mean	SD	Mean	SD
Pr(hypermethylation)					
* ABLIM1*	>0.99	0.14	0.11	−0.82	0.54
* DCC*	>0.99	0.14	0.16	−0.36	0.28
* EFNA2*	0.96	0.07	0.13	−0.05	0.22
* EFNB1*	0.98	0.07	0.08	−0.27	0.38
* EPHA5*	0.99	0.08	0.09	−0.12	0.30
* EPHA8*	0.96	0.06	0.07	0.09	0.23
* LIMK1*	0.99	0.10	0.12	0.36	0.24
* LRRC4*	0.98	0.11	0.15	−0.29	0.40
* NTNG1*	0.96	0.06	0.07	−0.67	0.47
* PAK2*	>0.99	0.13	0.13	−0.08	0.47
* PAK7*	0.97	0.08	0.12	0.08	0.13
* PLXNB1*	0.99	0.13	0.17	<0.01	0.13
* SEMA3G*	0.99	0.09	0.08	−1.72	1.35
* SEMA3E*	0.91	0.04	0.08	−1.22	1.47
* SLIT2*	0.93	0.05	0.10	−1.62	1.11
* SLIT3*	0.93	0.06	0.13	−0.44	0.38
* UNC5A*	0.92	0.06	0.13	0.06	0.21
* UNC5C*	0.91	0.05	0.11	−0.07	0.13
Pr(hypomethylation)					
* MAPK1*	>0.99	−0.15	0.14	−0.23	0.35
* HRAS*	0.97	−0.07	0.06	−0.04	0.41
* EPHA1*	0.98	−0.07	0.08	0.24	0.21
* NFATC2*	0.91	−0.05	0.05	−0.19	0.39
* NGEF*	0.91	−0.05	0.06	0.52	0.39
* NTNG2*	0.91	−0.06	0.12	−0.23	0.35
* PLXNB3*	0.96	−0.08	0.10	0.43	0.51
* PPP3R2*	0.98	−0.10	0.12	<0.01	0.11
* SEMA3B*	0.97	−0.10	0.15	−0.60	0.43
* SEMA4A*	0.98	−0.16	0.19	<0.01	0.61
* SEMA4G*	0.93	−0.05	0.06	0.25	0.51
* SEMA6B*	0.98	−0.10	0.11	−0.06	0.41

The corresponding average and standard deviation of difference in DNA methylation and gene expression are also listed.

**Table 4:** Genes with large probabilities of hypermethylation or hypomethylation in the two pathways in the HG-DCIS study.

P53 pathway		Difference in DNAm		Difference in Gene Exp	
Pr(hypermethylation)		Mean	SD	Mean	SD
* GTSE1*	0.91	0.20	0.11	2.65	0.83
* TSC2*	0.95	0.19	0.07	0.09	0.61
Pr(hypomethylation)					
* ADGRB1*	0.95	−0.29	0.10	–	–
* CASP3*	0.96	−0.31	0.11	−0.22	0.46
* CASP9*	0.91	−0.13	0.10	0.58	0.42
* CCND1*	0.91	−0.15	0.16	1.48	1.17
* CCND3*	0.94	−0.18	0.10	−0.06	0.51
* CDKN2A*	0.91	−0.15	0.14	1.34	0.86
* TP73*	0.93	−0.25	0.14	1.11	0.66
mTor pathway					
Pr(hypermethylation)					
* AKT3*	0.94	0.22	0.15	−1.87	0.83
* EIF4E2*	0.95	0.17	0.13	−1.10	0.56
* INS*	0.95	0.24	0.17	–	–
* PDPK1*	0.91	0.21	0.19	−0.04	0.39
* PIK3CB*	0.97	0.20	0.08	−0.60	0.63
* PIK3CD*	0.95	0.18	0.12	−0.39	1.28
* PIK3R3*	0.94	0.15	0.11	1.08	0.73
* PRKCA*	0.94	0.23	0.15	−0.50	1.21
* PRKCB*	0.95	0.18	0.07	3.45	1.77
* PRKCG*	0.95	0.18	0.12	–	–
* RPS6KA1*	0.92	0.12	0.08	−1.36	0.54
* RPS6KA2*	0.95	0.15	0.08	−1.22	0.71
* RPTOR*	0.97	0.23	0.10	0.04	0.77
* STK11*	0.96	0.20	0.11	−0.12	0.38
* TSC2*	0.95	0.19	0.07	0.09	0.61
Pr(hypomethylation)					
* PDPK1*	0.92	−0.20	0.06	−0.04	0.39
