# Supplementary material for: Gene-set Analysis with CGI Information for Differential DNA Methylation Profiling
Source: Sci Rep. 2016 Apr 19;6:24666. doi: 10.1038/srep24666 (PMC4836301; doi:10.1038/srep24666)
Supplement: Supplementary Information [file srep24666-s1.pdf]

Gene-set Analysis with CGI Information for Differential DNA Methylation Profiling

Chia-Wei Chang, Tzu-Pin Lu, Chang-Xian She, Yen-Chen Feng, Chuhsing Kate Hsiao

Supplementary Text 1.

This file contains information for model specification, data management, computational details, and codes in R for package R2OpenBUGS.

Notation and Models

Under a matched case-control study, let i represent the ith case-control pair recruited, and θ_{ij} denote the difference in DNA methylation levels between the case and the control at the jth probe (P_j). A large positive value of θ_{ij} indicates hypermethylation in cases compared to controls; while a large negative value implies hypomethylation. The indices i and j assume that the difference is probe-specific and pair-specific. The property of pair-specificity has been observed in earlier studies (Martino et al. 2013). That is, the difference in DNAm between each case-control pair per probe varies among probes. This difference follows a normal distribution $N(\mu_{ij}, \sigma_j^2)$ where both mean and variance parameters are probe specific. This assumption takes account of the heterogeneity across genes (indexed by j) and between individuals (indexed by i).

$$\theta_{ij} \mid \mu_{ij}, \sigma_j^2 \sim N(\mu_{ij}, \sigma_j^2) \quad \text{for every probe } j$$

To further decipher the possible sources of difference in DNAm, we next assume a

normal hyper-prior $N(\lambda_j, \sigma_\mu^2)$ for each μ_{ij}, where its mean parameter λ_j is determined by the sum of a CGI-dependent quantity β_j and a function $\Phi(\boldsymbol{\gamma}; P_j, \boldsymbol{\Lambda})$ of probe P_j and K given pathways $\boldsymbol{\Lambda} = \{\Lambda_1, \Lambda_2, ..., \Lambda_K\}$,

$$\mu_{ij} \mid \lambda_j, \sigma_\mu^2 \sim N(\lambda_j, \sigma_\mu^2)$$

$$\text{where } \lambda_j = \beta_j + \Phi(\boldsymbol{\gamma}; P_j, \boldsymbol{\Lambda}).$$

CGI Information β_j

The first component β_j in λ_j contains the effect of CGI status. The probes in a CGI may each exert a similar effect, which may differ from the effect exerted by non-CGI probes. Therefore, the β_j is distributed as,

$$\beta_j \sim I(P_j \notin \text{CGI}) \times N(0, \sigma_{\beta_1}^2) + I(P_j \in \text{CGI}) \times N(0, \sigma_{\beta_2}^2).$$

Those probes located in CGIs (i.e., $I(P_j \in \text{CGI}) = 1$) all share a similar effect with distribution $N(0, \sigma_{\beta_2}^2)$; while those not in CGIs $I(P_j \notin \text{CGI}) = 1$) share a similar effect with distribution $N(0, \sigma_{\beta_1}^2)$. These two distributions allow the variability of the effect to differ when CGI status differs.

Pathway Information $\Phi(\boldsymbol{\gamma}; P_j, \boldsymbol{\Lambda})$

The second component $\Phi(\boldsymbol{\gamma}; P_j, \boldsymbol{\Lambda})$ in λ_j contains the overall effect of the pathways $\{\Lambda_1, \Lambda_2, ..., \Lambda_K\}$ with corresponding parameters $\boldsymbol{\gamma} = (\gamma_1, \gamma_2, ..., \gamma_K)$, where each γ_k represents a pathway-specific effect. The specification of the function Φ is flexible. More importantly, this formulation allows a given gene to appear in multiple pathways, performing in each a different function or role (Luscombe et al. 2004). For

instance, this function can be $\sum_{k=1}^{K} f_k(P_j)$, the sum of $f_k\left(P_j\right)$, $k=1,\ldots,K$, where each f_k represents the information from each individual pathway as well as its constituents. If any cross-talk exists among pathways, then this can be incorporated in the function Φ. Several examples of the specification as well as the contribution from its gene members will be described in the following sections. The complete Bayesian hierarchical model for the differential DNA methylation profiling is

$$\begin{cases} \theta_{ij}|\mu_{ij},\sigma_j^2 \sim N(\mu_{ij},\sigma_j^2) \\ \mu_{ij} \mid \lambda_j,\sigma_\mu^2 \sim N(\lambda_j,\sigma_\mu^2) \\ \qquad \text{where } \lambda_j = \beta_j + \Phi(\boldsymbol{\gamma};P_j,\boldsymbol{\Lambda}) = \beta_j + \Phi(\gamma_1,\gamma_2,\ldots,\gamma_K;P_j,\Lambda_1,\Lambda_2,\ldots,\Lambda_K) \\ \beta_j \sim I(P_j \notin \text{CGI}) \times N(0,\sigma_{\beta_1}^2) + I(P_j \in \text{CGI}) \times N(0,\sigma_{\beta_2}^2) \\ \gamma_k \sim N(0,\sigma_\gamma^2) \\ \boldsymbol{\Sigma} = (\sigma_j^2,\sigma_\mu^2,\sigma_{\beta_1}^2,\sigma_{\beta_2}^2,\sigma_\gamma^2) \sim \pi(\cdot) \end{cases}$$

Here γ_k stands for the pathway effect and λ_j is used to evaluate and select influential genes. In addition, the impact of CGI on the DNAm is assessed through β_j. Those probes located in CGIs (i.e., $I(P_j \in \text{CGI}) = 1$) share a similar effect with distribution $N(0,\sigma_{\beta_2}^2)$; while for those not in CGIs ($I(P_j \notin \text{CGI}) = 1$) share an effect with distribution $N(0,\sigma_{\beta_1}^2)$. These two distributions allow the variability of effect to differ when CGI status differs. The prior distribution of γ_k is assumed to follow a Normal distribution with mean 0 and a constant variance σ_γ^2. For each of the variance parameters, σ_j^2, σ_μ^2, $\sigma_{\beta_1}^2$, $\sigma_{\beta_2}^2$, and σ_γ^2, we assume the Inverse-Gamma prior distribution.

Specification of Pathway Information $\Phi(\gamma;P_j,\boldsymbol{\Lambda})$

Suppose there are K pathways to be examined for possible association with the disease under study. Then, to incorporate such biological information, we propose the following functions for the following different scenarios. Suppose the probe P_j locates in gene G_j, which may or may not be in the kth pathway, where $k=1,\ldots,K$. To simplify the notation, we assume for now that the joint-effect Φ can be partitioned into $f_k\left(P_j\right)$, where $k=1,...,K$. That is, $\Phi(\boldsymbol{\gamma};P_j,\boldsymbol{\Lambda})=\sum_{k=1}^{K} f_k(P_j)$ as shown below. We now define the functions $f_k\left(P_j\right)$ in the following ways to account for the given type of pathway information.

(a) Null effect: $\Phi(\boldsymbol{\gamma};P_j,\boldsymbol{\Lambda})=0$.

The first way is for the "null effect". This represents the case where each of the K pathways has no impact on the phenotype. This can occur when each pathway shows no relationship with the level of DNAm. In other words, $f_k\left(P_j\right)=0$ for every k under the independence assumption for the K pathways. The null information can also stand for the case when the gene is not a member of the pathway.

(b1) CGI-independent constant pathway effect:

$$\Phi(\boldsymbol{\gamma};P_j,\boldsymbol{\Lambda})=\sum_{k=1}^{K} f_k\left(P_j\right), \text{ where } \quad f_k\left(P_j\right)=I_k(P_j)\times\gamma_k$$

The second example is for the case when all members in the same pathway share a constant effect, γ_k, which quantifies the influence of this pathway on DNA methylation changes. Here $I_k(P_j)$ denotes an indicator function with the value 1 if

gene G_j is a member of the kth pathway and 0 otherwise. If one gene G_j appears in multiple pathways, say Λ_{k_1} and Λ_{k_2}, then the corresponding γ_{k_1} and γ_{k_2} allow the same gene to play a different role and exert a different effect in these two pathways.

(b2) CGI-dependent constant pathway effect:

$$\Phi(\boldsymbol{\gamma}; P_j, \boldsymbol{\Lambda}) = \sum_{k=1}^{K} f_k\left(P_j\right), \text{ where } \quad f_k\left(P_j\right) = \begin{cases} I_k(P_j) \times \gamma_k^C, & \text{if } P_j \in \text{CGI} \\ I_k(P_j) \times \gamma_k^{nC}, & \text{if } P_j \notin \text{CGI} \end{cases}$$

If the role played by gene G_j in the pathway depends on whether the probe P_j is in a CGI or not, then the common constant pathway effect in (b1) should be categorized into two parts, one for those in CGIs, denoted γ_k^C, and one for those not in CGIs, denoted γ_k^{nC}. Comparison of these two quantities may reveal whether or not CGI status further modifies the role of G_j and the impact of the pathway.

(c1) CGI-independent degree effect:

$$\Phi(\boldsymbol{\gamma}; P_j, \boldsymbol{\Lambda}) = \sum_{k=1}^{K} f_k\left(P_j\right), \text{ where } \quad f_k\left(P_j\right) = E_{jk} \times \gamma_k$$

Let E_{jk} denote the number of neighboring genes of G_j in the k-th pathway. This number can provide information about whether or not this gene is a hub gene and about how influential it is in maintaining the integrity and normal function of the pathway. For instance, in network analysis the number of incoming and outgoing links per node (gene) is called the degree or connectivity, which can influence the performance of the network. In that case, the methylation level of gene G_j may depend on E_{jk}, and thus can modify γ_k. This information function, therefore, implies that the effect of each gene

member G_j in Λ_k is no longer constant within this pathway. In other words, each gene can exert extra influence on the phenotype of interest, in addition to the common pathway effect γ_k. In addition, such information about interconnectivity is not necessarily restricted to the number of links between genes. It can be physical protein interactions, gene co-expressions, or functional interactions derived from other computational models as well.

(c2) CGI-dependent degree effect:

$$\Phi(\boldsymbol{\gamma}; P_j, \boldsymbol{\Lambda}) = \sum_{k=1}^{K} f_k\left(P_j\right), \text{ where } \quad f_k\left(P_j\right) = \begin{cases} E_{jk} \times \gamma_k^C, \text{ if } P_j \in \text{CGI} \\ E_{jk} \times \gamma_k^{nC}, \text{ if } P_j \notin \text{CGI} \end{cases}$$

Similar to the case in (b2) for CGI-dependence, the degree effect may differ according to CGI status. That is, the quantity γ_k^C is for genes whose probes locate in CGIs and γ_k^{nC} for genes whose probes are not in CGIs.

Data management, matching, and normalization:

The ovarian cancer study from the United Kingdom Ovarian Cancer Population Study (UKOPS) recruited 266 postmenopausal women with ovarian cancer (131 pre-treatment and 135 post-treatment cases) and 274 age-matched healthy controls. The DNA methylation data are available from the GEO database (accession number GSE19711). The Illumina HumanMethylation27 BeadChip was used to obtain DNA methylation profiles across 27,578 CpGs in 540 whole blood samples. DNA was bisulphate converted, amplified, fragmented and hybridized to the BeadChip arrays. The methylation level of each specific CpG site was calculated from the intensity values of methylated and unmethylated DNA beads as a ratio of fluorescent signals. This ratio is

usually called the β value. It is continuous between 0, standing for completely unmethylated, and 1 for completely methylated.

The procedures for data management include outlier detection, removal of batch effect, matching, and normalization. First, the quality of data was evaluated based on methylation level, defined as the ratio of signal from a methylated probe relative to the sum of both methylated and unmethylated probes. As the first step, the outliers were detected with the boxplot of 540 ratio values per each CpG site. Any value larger or smaller than 1.5 times the IQR (inter-quartile range) was denoted as an outlier value. Next, for each sample subject, the number of CpG sites detected as outlier values among the total 27,578 sites was counted, and this number was defined as the "outlying number" for this subject sample. A new boxplot of such numbers of outliers for all 540 subjects was then created. Any extreme value in this new boxplot corresponds to a subject whose outlying number is extreme and thus this subject was considered as an outlying subject sample and removed from analysis. This excluded a total of sixty-two subjects, 11 of which were pre-treatment patients, 21 post-treatment patients, and 30 healthy controls.

After the quality control procedure, 478 subject samples remained. The quantile normalization was next performed with the preprocessCore package in R to avoid a potential batch effect, following the removal of three batches with an overwhelming portion of healthy samples (47 controls and 0 case in batches numbered 10 and 11; 19 controls and 4 cases in batch 12). For the remaining batches, the number of controls was between 15 and 21; while the number of cases was between 26 and 32. In addition, to avoid the interference of a treatment effect on methylation, we removed the 112 post-treatment cases for the remainder of the analysis. When the data

management procedure was finished, we were left with 118 pre-treatment cases and 135 controls. We then matched case-control pairs by restricting the difference in age to be less than three, obtaining 104 case-control pairs for the following analysis.

Computational details:

The final methylation data included 104 case-control pairs and 1,675 probes. The CpG island information was obtained from the identification information in Illumina Infinium 27k Human DNA methylation Beadchip v1.2 downloaded from the GEO database. The KEGG pathway information was obtained from the R package graphite version 1.8.1. Hyper-parameters in the prior distributions were set to $\sigma_{\mu}^{2} = 900^{-1}$, $\sigma_{\beta 1}^{2} = 600^{-1}$, $\sigma_{\beta 2}^{2} = 429^{-1}$, $\sigma_{\gamma}^{2} = 1500^{-1}$, $a = 0.05$, $b = 0.3$. The settings were based on Feng's approach (2012) and preliminary data analysis. For sensitivity analysis, we have tried several hyper-parameter settings. The results showed that the posterior distributions were robust to the settings of hyper-parameters.

All the parameters were estimated by the Markov chain Monte Carlo (MCMC) method using R2OpenBUGS package in R. After a burn-in of 1,000 iterations, every 1 in 10 posterior samples was collected to avoid correlation among samples. After 10,000 iterations, the final 1,000 posterior samples for each parameter were used for statistical analysis. The convergence of all iterations has been confirmed by MC errors and trace plots.

References

Feng, Ye-Chen (2012) Bayesian inference of DNA hypermethylation based on global methylation profiling, master thesis, Institute of Epidemiology and Preventive

Medicine, National Taiwan University.

Luscombe, N. M., Baby, M. M., Yu, H. et al. (2004) Genomic analysis of regulatory network dynamics reveals large topological changes, Nature, 431:308-312.

Martino, D. Loke, Y. J., Gordon, L. et al. (2013) Longitudinal, genome-scale analysis of DNA methylation in twins from birth to 18 months of age reveals rapid epigenetic change in early life and pair-specific effects of discordance, Genome Biology, 14:R42.

Codes in R package R2OpenBUGS:

```
# Things to do in R
# (a) install packages "R2OpemBUGS",
# (b) use the function "bugs" in this package after the following codes
#
# DATA
#     I = 104; number of case-control pairs
#     J = 1675; number of probes
#     K = 10; # number of pathways
#     theta.p[i, j]; difference of DNAm level for the ith case-control pair at jth probe
#     CGI.p[j]; 1: jth probe if in CGI, 0: if not in CGI
#     Ind.path.p[j, k]; 1: if jth probe contained in kth pathway, 0: if not
#     Edg.path.p[j, k]; (neighbor effect) number of edges of jth probe in kth pathway
#
# PARAMETERS
#     mu[i, j]; expected value of theta.p[i, j]
#     tau[j]; precision of theta.p[i, j]
#     sigma[j]; variance of theta.p[i, j]
#     lambda[j]; expected value of mu[i, j]
#     beta.nCGI[j]; effect of jth probe if locating outside CGI
#     beta.CGI[j]; effect of jth probe if locating in CGI
#     gamma[k]; effect of kth pathway
#     gamma.CGI[k]; effect of the probe if locating in CGI, in the kth pathway
#     gamma.nCGI[k]; effect of the probe if locating outside CGI
#     gamma.I[j, k]; effect of kth pathway for jth probe

# Null model (a)
model
{     for (j in 1:J) {
            for (i in 1:I) {
                  theta.p[i, j] ~ dnorm(mu[i, j], tau[j])
                  mu[i, j] ~ dnorm(lambda[j], 900)    }
             beta.nCGI[j] ~ dnorm(0.00000E+00, 600)
             beta.CGI[j] ~ dnorm(0.00000E+00, 429)
```

```
            tau[j] ~ dgamma(0.05, 0.3)
            sigma[j] <- 1/sqrt(tau[j])
            lambda[j] <- beta.nCGI[j] * (1 - CGI.p[j]) + beta.CGI[j] * CGI.p[j]    } }
# Common pathway effect (b1)
model
{      for (k in 1:K) {
            gamma[k] ~ dnorm(0.00000E+00, 1500)    }
      for (j in 1:J) {        for (i in 1:I) {
                  theta.p[i, j] ~ dnorm(mu[i, j], tau[j])
                  mu[i, j] ~ dnorm(lambda[j], 900)      }    }
      for (j in 1:J) {        for (k in 1:K) {
                  gamma.I[j, k] <- gamma[k] * Ind.path.p[j, k]      }
            beta.nCGI[j] ~ dnorm(0.00000E+00, 600)
            beta.CGI[j] ~ dnorm(0.00000E+00, 429)
            tau[j] ~ dgamma(0.05, 0.3)
            sigma[j] <- 1/sqrt(tau[j])
            lambda[j] <- beta.nCGI[j] * (1 - CGI.p[j]) + beta.CGI[j] * CGI.p[j] +
                  sum(gamma.I[j, 1:K])    }    }
# CGI-dependent pathway effect (b2)
model
{      for (k in 1:K) {
            gamma.CGI[k] ~ dnorm(0.00000E+00, 1500)
            gamma.nCGI[k] ~ dnorm(0.00000E+00, 1500)           }
      for (j in 1:J) {      for (i in 1:I) {
                  theta.p[i, j] ~ dnorm(mu[i, j], tau[j])
                  mu[i, j] ~ dnorm(lambda[j], 900)      } }
      for (j in 1:J) {      for (k in 1:K) {
                  gamma.I[j, k] <- gamma.CGI[k] * Ind.path.p[j, k] *
                        CGI.p[j] + gamma.nCGI[k] * Ind.path.p[j, k] *
                        (1 - CGI.p[j])             }
            beta.nCGI[j] ~ dnorm(0.00000E+00, 600)
            beta.CGI[j] ~ dnorm(0.00000E+00, 429)
            tau[j] ~ dgamma(0.05, 0.3)
            sigma[j] <- 1/sqrt(tau[j])
            lambda[j] <- beta.nCGI[j] * (1 - CGI.p[j]) + beta.CGI[j] * CGI.p[j] +
                  sum(gamma.I[j, 1:K])      } }
# Neighbor effect (c1)
# Inverse neighbor effect (modify Edg.path.p)
model
{      for (k in 1:K) {
            gamma[k] ~ dnorm(0.00000E+00, 1500)         }
      for (j in 1:J) {        for (i in 1:I) {
                  theta.p[i, j] ~ dnorm(mu[i, j], tau[j])
                  mu[i, j] ~ dnorm(lambda[j], 900)    }    }
      for (j in 1:J) {        for (k in 1:K) {
                  gamma.I[j, k] <- gamma[k] * Edg.path.p[j, k]      }
            beta.nCGI[j] ~ dnorm(0.00000E+00, 600)
            beta.CGI[j] ~ dnorm(0.00000E+00, 429)
            tau[j] ~ dgamma(0.05, 0.3)
```

```
sigma[j] <- 1/sqrt(tau[j])
lambda[j] <- beta.nCGI[j] * (1 - CGI.p[j]) + beta.CGI[j] * CGI.p[j] +
        sum(gamma.I[j, 1:K])      } }
```

Table S1: Scores of Strength

The first column contains the strength score of each competing pathway. Other columns contain numbers that are 2 times the absolute difference between the posterior probability $\Pr(\gamma_k > 0 \mid y, \text{ model } M)$ and 0.5. For models (b2) and (c2), the max among Y and N was used. The range of all values is (0, 1). Larger values indicate stronger effects.

**KEGG pathway term (no. of genes/no. of probes)**	**Strength score**	**Constant effect model**			**Degree effect model**		
		(b1) CGI-independent	(b2) CGI-dependent		(c1) CGI-independent	(c2) CGI-dependent	
			Y	N		Y	N
hsa05200 Pathways in cancer (303/714)	0.84	0.97	0.96	0.94	0.62	0.81	0.30
hsa04110 Cell cycle (120/308)	0.83	0.91	0.94	0.12	0.69	0.77	0.09
hsa04114 Oocyte meiosis (101/211)	0.62	0.57	0.71	0.31	0.54	0.64	0.12
hsa00980 Metabolism of xenobiotics by cytochrome P450 (69/122)	0.39	0.40	0.38	<0.01	0.12	0.66	0.48
hsa00330 Arginine and proline metabolism (53/100)	0.27	0.11	0.50	0.39	0.02	0.46	0.37
hsa04115 p53 signaling pathway (67/201)	0.34	0.37	<0.01	0.24	0.14	0.37	0.60
hsa04610 Complement and coagulation cascades (64/113)	0.34	0.14	0.49	0.17	0.18	0.53	0.08
hsa00982 Drug metabolism - cytochrome P450 (63/111)	0.24	0.24	0.09	0.07	0.17	0.32	0.44
hsa00350 Tyrosine metabolism (31/57)	0.18	0.02	0.14	0.13	0.05	0.52	0.09
hsa04510 Focal adhesion (192/418)	0.11	<0.01	0.11	0.38	0.02	0.11	0.18

Table S2: Gene with large probabilities of hypermethylation and hypomethylation among tumor tissues under three pathways in the lung cancer study.

**GNRH**		**Prolactin**		**Glycosphingolipid**	
**Pr(hypermethylation)**					
ADCY1	0.95	*CCND1*	0.97	*B3GALT2*	0.99
ADCY2	0.99	*ELF5*	0.96	*FUT9*	0.93
ADCY4	0.94	*ESR1*	0.99	*GCNT2*	0.94
ADCY5	0.97	*LHCGR*	0.95		
ADCY8	0.96	*MAP2K2*	0.94		
ADCY9	0.93	*PRL*	0.98		
CACNA1S	0.91	*RELA*	0.95		
EGFR	0.91	*SOCS2*	0.96		
GNAS	0.94	*STAT5A*	0.96		
GNRH2	0.93	*STAT5B*	0.94		
MMP14	0.94				
MMP2	0.97				
MAP2K2	0.94				
**Pr(hypomethylation)**					
HRAS	0.97	*AKT3*	0.98	*ABO*	0.98
CACNA1S	0.99	*CCND1*	0.98	*B3GALT1*	>0.99
CALML3	0.94	*HRAS*	0.97	*B3GALT5*	0.97
FSHB	0.99	*INS*	0.98	*B3GNT3*	0.98
ITPR2	0.93	*MAPK1*	>0.99	*FUT2*	0.99
MAPK1	>0.99	*MAPK13*	0.99	*FUT3*	0.98
MAPK13	0.99	*SHC3*	0.95	*FUT6*	0.99
MAP2K3	0.99	*SOCS1*	>0.99	*FUT7*	0.92
PLA2G4C	0.94	*SRC*	0.96		
PLA2G4E	0.91	*STAT5A*	0.91		
PLCB2	0.98	*TNFSF11*	0.99		
PLCB4	0.94				
SRC	0.96				

Figure S1a : Boxplots of 200 randomly selected probes (θ_{ij}) locating in CGIs.

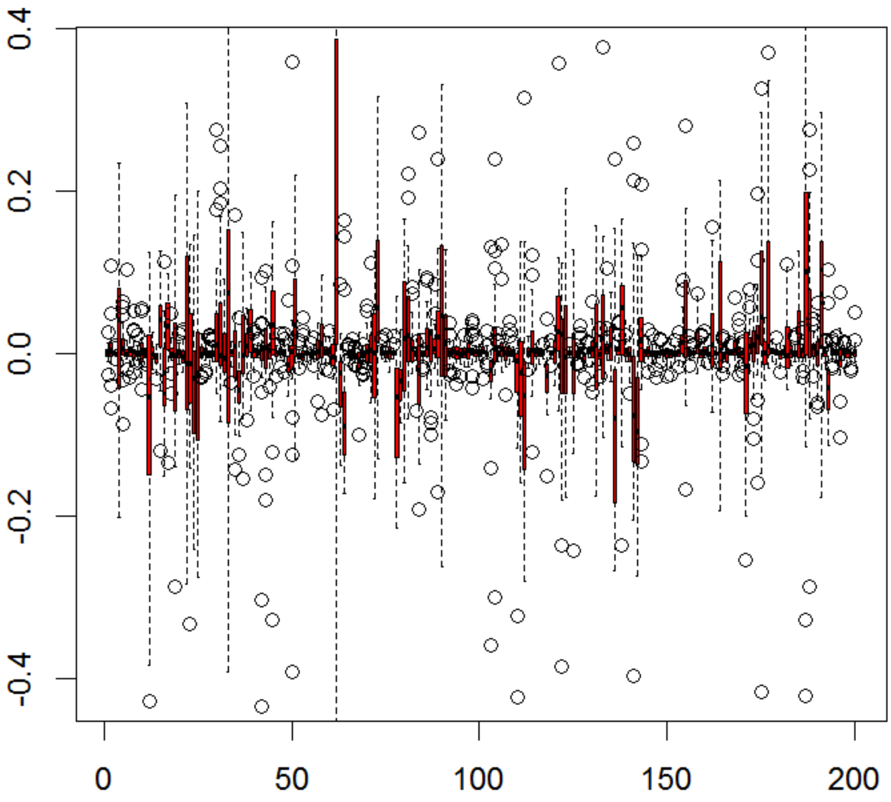

Figure S1b: Boxplots of 200 randomly selected probes (θ_{ij}) locating outside CGIs.

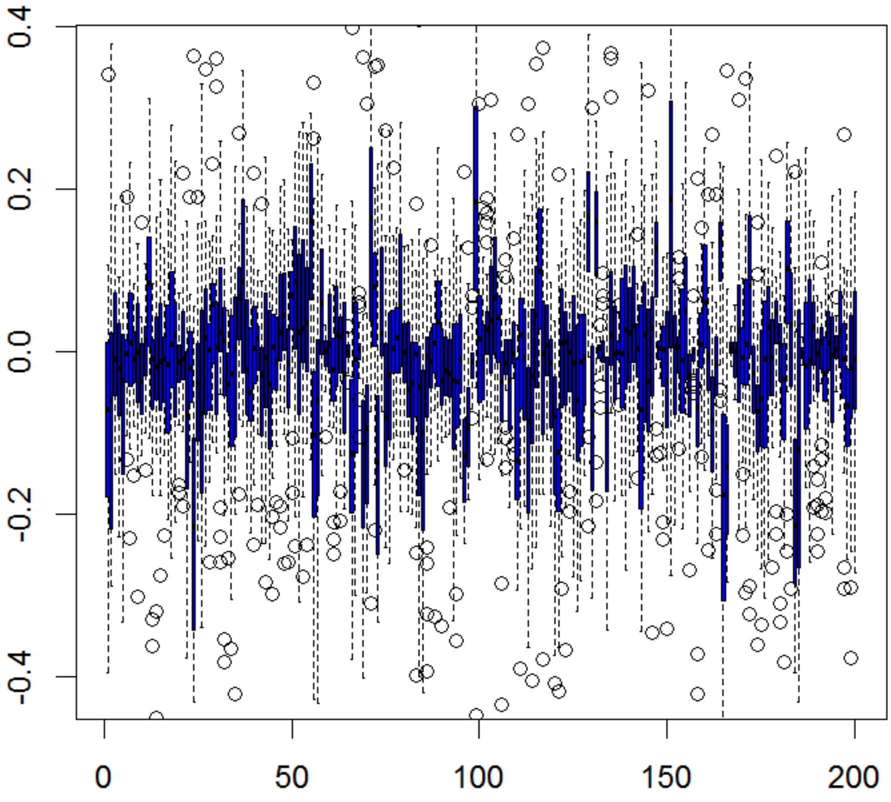

Figure S2: To visualize the difference between the DMGs identified in model (b2) and the disease-related genes reported in other studies, we colored the genes in the plot of Pathways in cancer in Figure S2. If the DMGs were also reported in more than one earlier study, they were colored in purple. If the DMGs were not covered by previous reports or were reported in only one article, then they were colored in blue. For genes not identified in model (b2) but in other studies, they were colored in darker pink if identified in methylation-related studies, or lighter pink if in non-methylation-related studies. Notice that, as compared with other genes in this pathway, the genes colored in blue are highly connected to the genes in purple or darker pink, and are more heavily linked among themselves. This pattern suggests not only significant association among blue genes, purple genes and the disease, but also a strong relationship between genes of these three colors (purple, blue and darker pink). These results can be considered as supplementary findings to earlier research and studied for further evaluation of their molecular interactions.

PATHWAYS IN CANCER

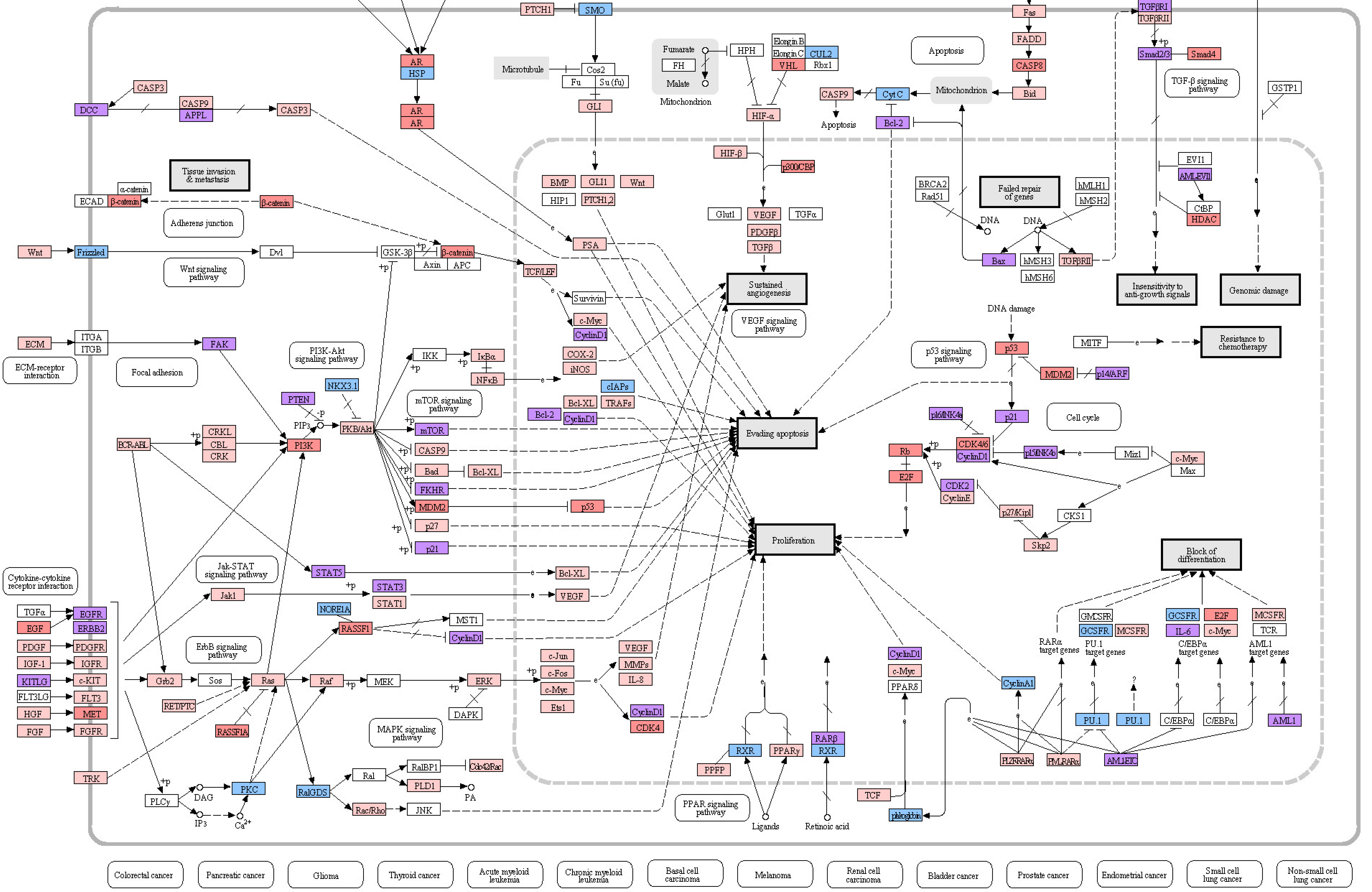

05200 8/7/13
(c) Kanehisa Laboratories
